# Ag-doping regulates the cytotoxicity of TiO_2_ nanoparticles *via* oxidative stress in human cancer cells

**DOI:** 10.1038/s41598-017-17559-9

**Published:** 2017-12-15

**Authors:** Maqusood Ahamed, M. A. Majeed Khan, Mohd Javed Akhtar, Hisham A. Alhadlaq, Aws Alshamsan

**Affiliations:** 1 King Abdullah Institute for Nanotechnology, King Saud University, Riyadh, Saudi Arabia; 2 Department of Physics and Astronomy, College of Science, King Saud University, Riyadh, Saudi Arabia; 3 Nanomedicine Research Unit, Department of Pharmaceutics, College of Pharmacy, King Saud University, Riyadh, Saudi Arabia

## Abstract

We investigated the anticancer potential of Ag-doped (0.5–5%) anatase TiO_2_ NPs. Characterization study showed that dopant Ag was well-distributed on the surface of host TiO_2_ NPs. Size (15 nm to 9 nm) and band gap energy (3.32 eV to 3.15 eV) of TiO_2_ NPs were decreases with increasing the concentration of Ag dopant. Biological studies demonstrated that Ag-doped TiO_2_ NP-induced cytotoxicity and apoptosis in human liver cancer (HepG2) cells. The toxic intensity of TiO_2_ NPs was increases with increasing the amount of Ag-doping. The Ag-doped TiO_2_ NPs further found to provoke reactive oxygen species (ROS) generation and antioxidants depletion. Toxicity induced by Ag-doped TiO_2_ NPs in HepG2 cells was efficiently abrogated by antioxidant N-acetyl-cysteine (ROS scavenger). We also found that Ag-doped TiO_2_ NPs induced cytotoxicity and oxidative stress in human lung (A549) and breast (MCF-7) cancer cells. Interestingly, Ag-doped TiO_2_ NPs did not cause much toxicity to normal cells such as primary rat hepatocytes and human lung fibroblasts. Overall, we found that Ag-doped TiO_2_ NPs have potential to selectively kill cancer cells while sparing normal cells. This study warranted further research on anticancer potential of Ag-doped TiO_2_ NPs in various types of cancer cells and *in vivo* models.

## Introduction

Wide-spread application of TiO_2_ nanoparticles (NPs) have been increasing due to their chemical stability, photocatalytic efficiency and low cast^1^. The TiO_2_ NPs are being utilized in daily life products such as sunscreens, paints and plastics^2^. Due to ever increasing market demand the annual production of TiO_2_ NPs is predicted to reach around 2.5 million tons by 2025^3^. There is also growing interest of TiO_2_ NPs in biomedical fields including drug delivery, cell imaging, photodynamic therapy and biosensor^4–6^. However, investigations have shown the conflicting results regarding the biological response of TiO_2_ NPs. Several studies found that TiO_2_ NPs induce inflammation, cytotoxicity and genotoxicity^7–9^. Contrary, several reports showed that TiO_2_ NPs were not toxic or least toxic to several cell lines^10–12^. Conflicting reports on toxicological response of TiO_2_ NPs could be due to utilization of different physical and chemical properties of this material^2,13^. In general, anatase and rutile are two crystalline forms of TiO_2_. Anatase TiO_2_ NPs have high photocatalytic activity and more biologically active than those of rutile one^14,15^.

Photocatalytic activity of TiO_2_ NPs is thoroughly investigated because of their applications in solar energy, environmental remediation and photodynamic therapy (PDT)^16,17^ since its breakthrough in 1980 s^18^. Under light irradiation, the valence band electrons (e^−^) of TiO_2_ become excited and moved to conduction band leaving positive charge holes (h^+^). The electrons (e^−^) in conduction band and holes (h^+^) in valence band have the capability to generated cellular reactive oxygen species (ROS)^19,20^. Light induced ROS generation by a photosensitizer has been applied in treatment of several diseases called PDT^21,22^. Potential of TiO_2_ NPs to be applied in PDT for different types of cancers, such as leukemia, cervical, liver and lung cancers is already reported^23,24^. Still, there are some drawbacks in the application of TiO_2_ NPs for PDT. The major drawbacks of TiO_2_ are wide band gap (3.2 eV for anatase) that can activate only in the ultraviolet (UV) region and high rate of electrons-holes (e^−^/h^+^) recombination that reduce considerably the photocatalytic efficiency of TiO_2_ NPs^25,26^.

Recent studies have now focused on the improvement of photocatalytic activity of TiO_2_ NPs. Attempts to achieve this goal is depends on doping of TiO_2_ NPs with metallic or non-metallic elements^27,28^. Doping can reduce the band gap of TiO_2_ NPs that extend their spectral response in visible wavelengths^29^. For example, doping of TiO_2_ NPs with noble metals such as Ag, Au or Pt can efficiently decrease the e^−^/h^+^ pair’s recombination to enhance the photocatalytic activity and simultaneously extend their light response towards the visible region because of their d electron configuration^30^. Among these Ag-doped TiO_2_ NPs has been thoroughly studied because of the dual function of Ag sites. First, Ag serves as an electron scavenging center to separate e^−^/h^+^ pairs because its’ Fermi level is below the conduction band of TiO_2_^30,31^. Second, Ag NPs have the ability to create surface plasmon resonance (SPR) effect of TiO_2_ NPs, thus leading to the distinctly enhanced photocatalytic activity of TiO_2_ NPs in visible region. However, application of Ag-doped TiO_2_ NPs in cancer therapy is not explored yet.

ROS generating potential of Ag-doped TiO_2_ NPs under visible light have been recently investigated in killing of microbial communities^32,33^. However, some studies have shown that Ag-doped TiO_2_ can kill bacteria without any light illumination^34,35^. This could be possible because Ag-doping tunes band gap (e^−^/h^+^ recombination) of TiO_2_ NPs that enhances the catalytic activity to generate ROS within bacterial cells without light illumination. Therefore, ROS generating potential of Ag-doped TiO_2_ NPs can be applied in treatment of cancer without the illumination of any light. Manipulating intracellular ROS level by redox modulators is a possible way to harm cancer cells selectively without affecting the normal cells^36–39^. Therefore, we explored the anticancer potential of Ag-doped TiO_2_ NPs via ROS pathway. Using Ag-doped TiO_2_ NPs without light in the treatment of cancer have some advantages over PDT^40^. For example, visible light used in PDT cannot travel very far through body tissue. Therefore, PDT is used to treat to the problem on or just under the skin on the lining of some internal organs or cavities. Metastasized cancer also cannot treat with PDT due to the inability of the light source to penetrate large tumors or reach areas where cancer may have spread. Hence, Ag-doped TiO_2_ NPs can have advantage of other exposure routes such as oral or intravenous injection. In this study, we investigated the cytotoxicity mechanisms of Ag-doped TiO_2_ NPs in human liver cancer (HepG2) cells. To avoid cell type-specific response we have also employed human lung (A549) and breast cancer (MCF-7) cells to assess the anticancer effect of Ag-doped TiO_2_ NPs. We have chosen these cancer cell lines because of the lung, liver and breast cancers are life menacing disease and the occurrence of these types of cancer are increasing rapidly worldwide^41–43^. These cell lines are also well-known *in vitro* models and have been widely utilized in toxicology and pharmacology studies^44–46^. We have also examined the benign nature of Ag-doped TiO_2_ NPs on two non-cancerous normal cells; human lung fibroblasts (IMR-90) and primary rat hepatocytes. We observed that Ag-doped TiO_2_ NPs selectively kill the cancer cells (HepG2, A549 & MCF-7) without much affecting the normal cells.

## Materials and Methods

### Preparation of nanoparticles

Pure and Ag-doped TiO_2_ NPs were synthesized by sol-gel procedure. Titanium (IV) isopropoxide Ti[OCH(CH_3_)_2_]_4_ and silver nitrate (AgNO_3_) were utilized as precursors. In brief, 0.1 M solution of titanium (IV) isopropoxide was prepared in absolute ethanol. Then, solution was mixed with distilled water and stirred for 2 h to get a clear and transparent TiO_2_ solution. The solution was further dried at 100 °C for 48 h to obtain TiO_2_ gel. After aging 24 h the TiO_2_ gel was filtered and dried. Then, prepared TiO_2_ samples were calcined at 400–600 °C for 24 h to get TiO_2_ nanopowder. The Ag-doped TiO_2_ nanopowder was synthesized by the same method as described above. The only difference was the addition of AgNO_3_ into the TiO_2_ solution. The dopant Ag concentrations were varied to 0.5, 2.5 and 5.0%, respectively.

### Characterization of nanoparticles

Crystal structure and phase purity of pure and Ag-doped TiO_2_ NPs were assessed by X-ray diffraction (XRD) (PanAnalytic X’Pert Pro) using Cu-K_α_ radiation (λ = 0.15405 nm, at 45 kV and 40 mA). Morphology was examined by field emmission transmission electron microscopy (FE-TEM) (JEM-2100F, JEOL Inc. Japan). Energy dispersive X-ray spectroscopy (EDS) was used to determine the elemental composition. Prepared NPs were also characterized micro-Raman spectroscopy through Horiba Raman system (IY-Horiba-T64000). UV-visible absorption spectra were obtained using a spectrometer (Shimadzu-2550, Japan). Surface composition and oxygen vacancies of the Ag-doped TiO_2_ NPs were determined by X-ray photoelectron spectroscopy (XPS) (PHI-5300 ESCA PerkinElmer, Boston, MA). The peak positions were internally referenced to the C 1 s peak at 284.6 eV. Aqueous behaviour (hydrodynamic size and zeta potential) of prepared NPs was assessed in a ZetaSizer Nano-HT (Malvern Instruments, UK).

### Cell culture and exposure of nanoparticles

The HepG2, A549, MCF-7 & IMR-90 cell lines were bought from American Type Culture Collection (ATCC) (Manassas, VA). Primary hepatocytes were isolated from rat using collagenase perfusion method^47^.

The DMEM medium supplemented with 10% fetal bovine serum (FBS) and 100 U/ml penicillin-streptomycin was used to culture the MCF-7 cells at 5% CO_2_ and 37 °C. At 80–90% confluence, cells were harvested sub-cultured for nanotoxicity parameters. Cells were allowed to attach on the surface of culture flask for 24 h prior to exposure of NPs. Pure and Ag-doped TiO_2_ NPs were suspended in DMEM medium and diluted to different concentrations (0.5–200 µg/ml). The NPs suspensions were then sonicated at room temperature for 10 min at 40 W to avoid agglomeration of NPs before exposure to cells. In some parameters, cells were pre-exposed for 1 h with N-acetyl-cysteine (NAC) (10 mM) before co-exposure with or without NPs. Hydrogen peroxide (H_2_O_2_) (2 mM), buthionine sulphoximine (BSO) (200 µM) or ZnO NPs (50 µg/ml) were also used as positive controls.

### Assay of cytotoxicity endpoints

Cell viability against NPs exposure was assessed by MTT and NRU assays. MTT assay was performed according to the protocol of Mossman^48^ with some modifications^49^. MTT assay assesses the function of mitochondrial by measuring the potential of living cells to reduce colorless MTT into blue formazon. The formazan was dissolved in acidified isopropanol and absorbance was recorded at 570 nm using a microplate reader (Synergy-HT, BioTek). Lysosomal activity (NRU cell viability assay) was performed according to the method of Borenfreund and Puerner^50^ with some modifications^51^. Cell membrane damage after NPs exposure was examined by lactate dehydrogenase (LDH) assay. LDH is an enzyme extensively found in the cytosol that converts lactate to pyruvate. Upon cell membrane damage, LDH leaks into extracellular matrix (culture medium). LDH level in culture medium was examined using a BioVision kit (Milpitas, CA). Morphology of cells after exposure to NPs was determined by phase-contrast inverted microscope (Leica) at 10X magnification.

### Assay of apoptotic markers

Mitochondrial membrane potential (MMP) was measured using Rh-123 fluorescent dye according to Siddiqui *et al*.^46^. MMP level was determined by two methods; cell imaging by fluorescent microscopy (OLYMPUS CKX 41) and quantitative assay by microplate reader (Synergy-HT, BioTek). Caspase-3 enzyme activity was determined by BioVision kit (Milpitas, CA). This assay is based on the principle that activated caspases in apoptotic cells cleave the synthetic substrates to release free chromophore p-nitroanilide (pNA)^52^. Cell cycle phases were measured by a Beckman Coulter Flow cytometer (Coulter Epics XL/Xl-MCL) through a FL-4 filter (585 nm) using propiodium iodide (PI) probe^53^. The data were analyzed by Coulter Epics XL/XL-MCL, System II Software.

### Assay of oxidative stress markers

Intracellular reactive oxygen species (ROS) generation was assessed utilizing 2,7-dichlorofluorescin diacetate (DCFH-DA) probe as reported elsewhere^54^ with few changes^46^. ROS level was determined by two methods; quantitative assay by a microplate reader (Synergy-HT, BioTek, USA) and cell imaging by fluorescent microscopy (OLYMPUS CKX 41). For the measurement of glutathione (GSH) level and superoxide dismutase (SOD) enzyme activity, cell extracts were prepared from the control and treated cells as described earlier^51^. Intracellular GSH level was quantified by Ellman‘ method^55^ using 5,5-dithio-bis-2-nitrobenzoic acid (DTNB). SOD enzyme activity was measured by a kit (Cayman Chemical Company, Michigan, OH).

### Protein estimation

Protein level was estimated by Bradford method^56^ using bovine serum albumin as standard.

### Statistics

One-way analysis of variance followed by Dunnett’s multiple comparison tests were performed for statistical analysis. Significance was ascribed at p < 0.05.

## Results and Discussion

### TEM analysis

**Figure 1:**
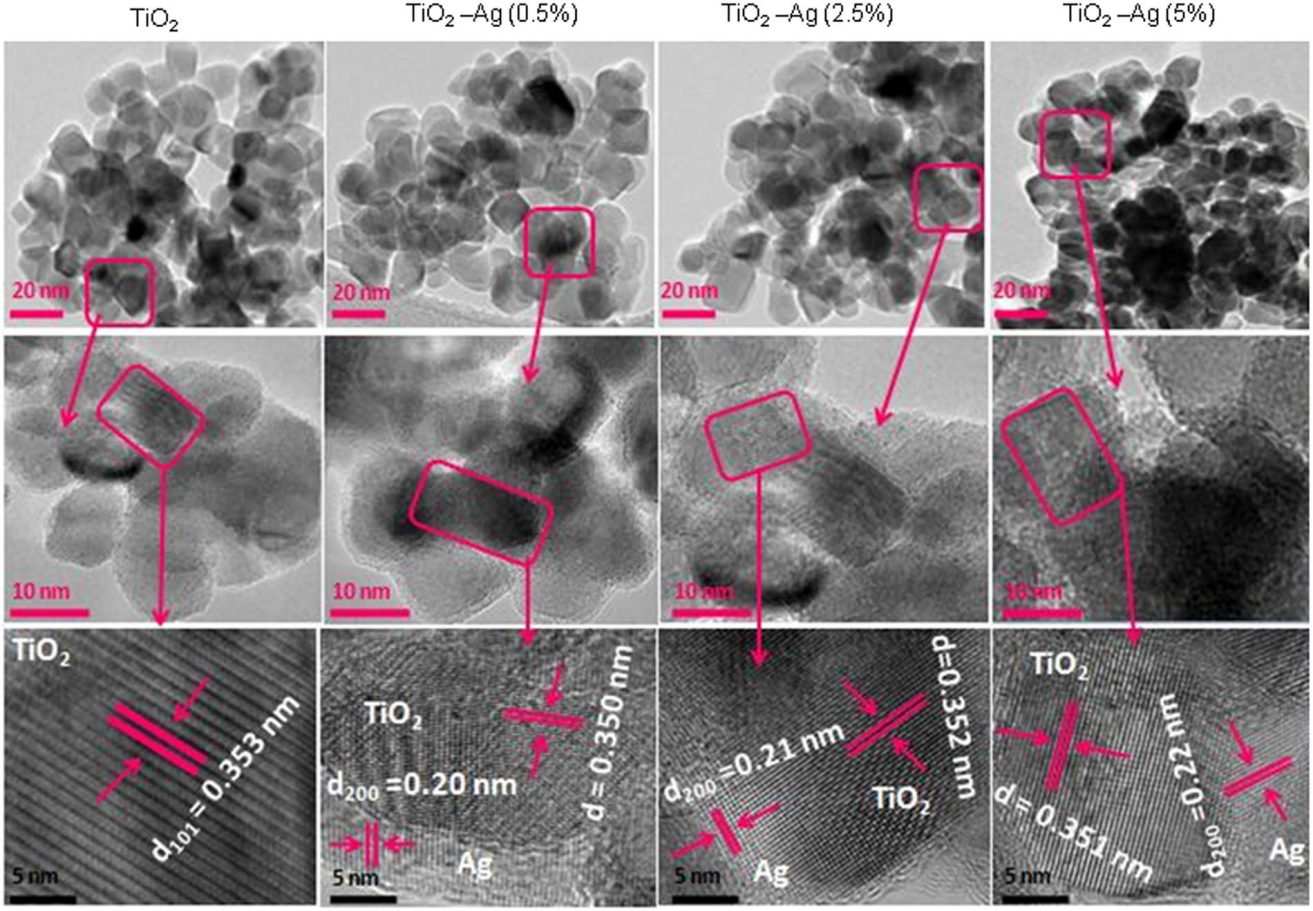
Field emission transmission electron microscopy (FETEM) characterization of pure and Ag-doped TiO_2_ NPs. Upper and middle panels represent low resolution images while lower panel presents the high resolution images of NPs.

Morphology and structural characterization of pure and Ag-doped TiO_2_ NPs were assessed by field emission transmission electron microscopy (FETEM) (Fig. 1). Upper and middle panels of Fig. 1 show low magnification images of pure and Ag-doped TiO_2_ NPs. The average particle size of pure TiO_2_ NPs was around 15 nm while particle size of Ag-doped (5%) TiO_2_ NPs was approximately 9 nm. These results indicated that Ag-doping reduces the size of host TiO_2_ NPs. Generally, metal ions doping at optimal level hinders the particles growth. Effect of Ag dopant on TiO_2_ NPs size reduction has been attributed to grain-boundary pinning caused by dopant ions, which limits the grain growth by the symmetry-breaking effects of the dopant at the boundary, resulting in smaller size of particles^57^. Reduction in size of NPs after doping was also reported in other studies^49,57^.

**Figure 2:**
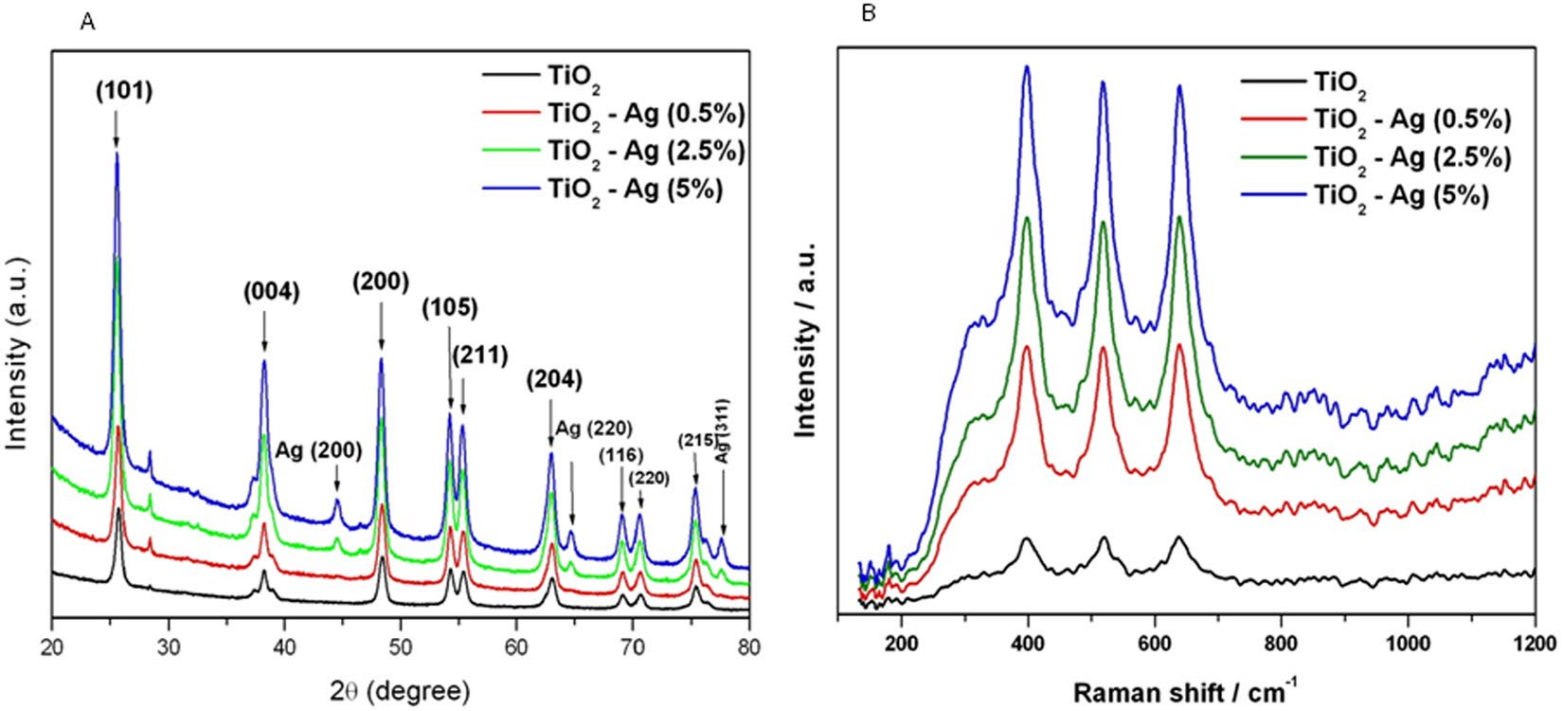
X-ray diffraction (XRD) and Raman spectroscopy characterization of pure and Ag-doped TiO_2_ NPs. (**A**) XRD spectra and (**B**) Raman spectra.

High resolution TEM images (lower panel of Fig. 1) clearly shows that dopant Ag was well distributed and decorated on the surface of host TiO_2_ NPs. High resolution TEM images also demonstrated that TiO_2_ NPs has a high crystalline nature with the plane spacing of 0.353 nm, 0.350 nm, 0.532 nm 0.351 nm, which matches well with (101) plane of anatase TiO_2_. After the combination with different amount of Ag (0.5, 2.5 & 5%), spacing between two adjacent lattice places is about 0.20, 0.21 and 0.22 nm, which corresponds to the (200) lattice distance of Ag (JCPDS: 04-0783). These lattice parameters were in agreement with the X-ray diffraction (XRD) spectra as shown in Fig. 2A.

### XRD analysis

XRD measurements were carried out to examine the crystallographic structure of prepared NPs. Figure 2A shows the XRD spectra of pure and Ag-doped (0.5–5%) TiO_2_ NPs. Diffraction peaks positioned at 2θ of 25.61°, 38.22°, 48.36°, 54.28°, 55.37°, 63.02°, 69.16°, 70.56° & 75.41° corresponds to the pure anatase phase of TiO_2_ (JCPDS No. 21–1272) and were assigned to do (101) (004), (200), (105), (211), (204), (116), (220) & (215) crystallographic planes. Moreover, the typical diffraction peaks (200), (220) & (311) positioned at 44.59, 64.70 & 77.57 indicates the face-centered cubic metallic Ag crystal structure (JCPDS 87-0597). Presence of Ag peaks suggested that Ag NPs are presented on the surface of TiO_2_ NPs. The broadening of the reflection peaks indicates small grains of prepared NPs. The average crystallite size of pure and Ag-doped TiO_2_ NPs were calculated by Scherrer equation^57^ using the full width at half maximum (FWHM) of the (101) diffraction peak. In agreement with TEM data, XRD also suggested that crystallite diameter of TiO_2_ NPs reduces with increasing the amount of Ag-doping.

### Raman analysis

Figure 2B represents the Raman spectra of pure and Ag-doped (0.5–5%) TiO_2_ NPs in the range 100–1200 cm^−1^ at room temperature. Three peaks with strong intensities are observed around 397 (B1g), 515(A1g), and 637 (Eg) cm^−1^, which indicates that all samples were mostly dominated by anatase phase of TiO_2_ NPs^58,59^. An interesting observation was that the peak intensities increased with the deposition of Ag, while the position of the Raman signal remained the same, indicating the crystallinity becomes better, which also corresponds to the results of XRD and high resolution TEM.

### XPS analysis

**Figure 3:**
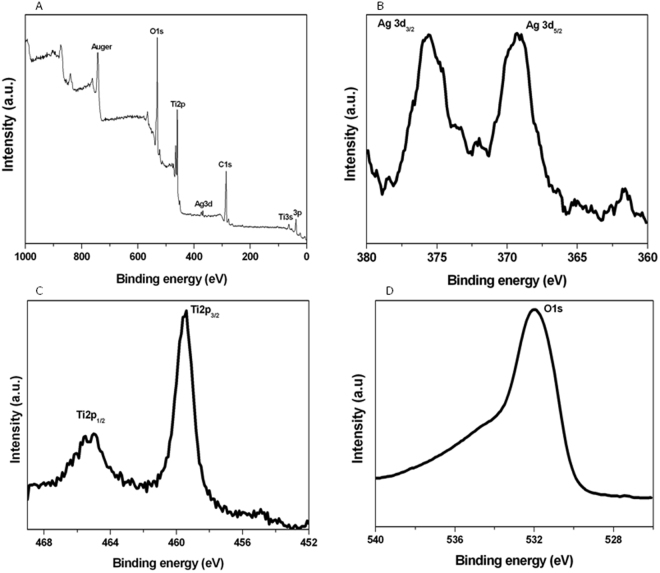
X-ray photoelectron spectroscopy (XPS) analysis of Ag-doped TiO_2_ NPs. (**A**) Survey, spectra in the (**B**) Ag3d, (**C**) Ti2P and (**D**) O1s regions of Ag-doped (5%) TiO_2_ NPs.

X-ray photoelectron spectroscopy (XPS) was performed to further characterize chemical composition and elemental status of pure and Ag-doped TiO_2_ NPs. Figure 3A shows the typical XPS survey spectra of Ag-doped (5%) TiO_2_ NPs. Results showed that Ti, O and Ag elements exist in Ag-doped TiO_2_ NPs. Peak located at binding energy of 463.75 eV corresponds to the Ti (2p1/2) and another one located at 460.12 eV is assigned to the Ti (2p3/2) (Fig. 3C). In the O1s region, highest intense peak at 529.8 eV is attributed to the lattice oxygen (Ti-O-Ti) in anatase (Fig. 3D). The Ag3d3 and Ag3d5 peaks indicated the presence of Ag in Ag-doped TiO_2_ NPs (Fig. 3B). The binding energies of Ag3d3 and Ag3d5 peaks are 369.5 eV and 371.4 eV, respectively. Our results have a strong agreement with the previous studies^60,61^. The EDS data also showed that Ti and O were the main elemental species in pure TiO_2_ NPs while additional Ag peaks were observed in Ag-doped TiO_2_ NPs supporting XPS results (Supplementary Fig. S1).

### Optical analysis

**Figure 4:**
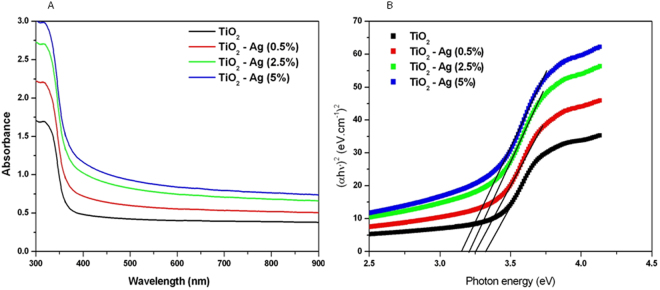
Optical characterization of pure and Ag-doped TiO_2_ NPs. (**A**) UV-visible absorption spectra and (**B**) (αhν)^2^ vs photon energy plots to determine band gap energy level.

Pure and Ag-doped TiO_2_ NPs absorption spectra are given in Fig. 4A. These absorption spectra indicated that there is red shift of the light absorption edge of Ag-doped TiO_2_ NPs in comparison to pure TiO_2_ anatase and the level of red shift increases with increasing the concentrations of Ag. The red shift of light absorption is the consequences of reduction in band gap energy. This is due to the lower Fermi level of Ag than those of TiO_2_. The shifting of light absorption edge of metal oxide NPs after metal ion doping was also reported in other studies^49,57^. Tauc Model was employed to determine the optical band gap energy of the aggregates, according to the following equation^49^:

$$\alpha hv=A{(h\nu -{E}_{g})}^{m}$$

where hν is the photon energy, E_g_ is the optical band gap, A is a constant, m is equal to 1/2 for allowed direct optical transitions and α is the absorption coefficient. The band gap values were determined by extrapolating the linear region of the plot to hν = 0. From the Tauc plots of (αhν)^2^ versus hν, the direct band gap values were estimated corresponding to 3.32 eV, 3.25 eV,3.20 eV and 3.15 eV for TiO_2_, Ti_99.5_Ag_0.5_O, Ti_97.5_Ag_2.5_O and Ti_95_Ag_5_O respectively (Fig. 4B). We can see that band gap energy (Eg) of TiO_2_ NPs decreases from 3.32 eV to 3.15 eV with increasing the level of Ag-doping. Reduction in band gap energy of semiconductor metal oxide NPs after doping with metal ions is also reported by other investigators^61,62^.

### Hydrodynamic size and zeta potential

It is essential to characterize the behavior of NPs in aqueous state before their biological studies. We have assessed the zeta potential and particle size of pure and Ag-doped TiO_2_ NPs in water and DMEM to get a realistic overview of NPs interaction with cells. We found that hydrodynamic size of pure and Ag-doped (0.5–5%) TiO_2_ NPs was 10–15 time higher than those of sizes calculated from TEM and XRD (primary particle size) (Table 1). We further noticed that hydrodynamic size of TiO_2_ NPs was slightly increases with the incremental of Ag-doping. Higher hydrodynamic size than primary particle size was also reported in other studies^63,64^. In ZetaSizer measurements higher size of NPs was because of tendency of NPs to agglomerate. We further observed little variation in hydrodynamic size of NPs dispersed in DMEM than those of deionized water. This could be due to presence of serum in the culture medium. It is known that serum could bind to NPs and form a protein corona^65^. This protein corona might be responsible for size variation in water and cell culture medium^66^. Protein corona presents on the surface of NPs also influences the interaction of NPs with cells. Zeta potential study suggested that pure and Ag-doped TiO_2_ NPs suspended in water had positive charge on the surface, whereas in culture medium NPs had negative surface (Table 1). Differences in surface charge could be due to adsorption of negative charged proteins on the surface of NPs.

**Table 1:** Structural and electronic properties of prepared nanoparticles.

Nanoparticles	TEM size (nm)	XRD size (nm)	Hydrodynamic size (nm)^#^		Zeta potential (mV)^#^		Band gap (eV)
			DI Water	CDMEM	DI Water	CDMEM	
TiO_2_	15.3 ± 1.7	14.8 ± 1.8	113.3 ± 8.6	142.5 ± 5.7	+21.5 ± 0.9	−17.2 ± 1.1	3.32
TiO_2_-Ag (0.5%)	14.4 ± 1.5	13.9 ± 1.6	126.5 ± 7.8	159.8 ± 6.5	+19.7 ± 1.5	−18.5 ± 1.3	3.25
TiO_2_-Ag (2.5%)	12.3 ± 1.3	12.1 ± 1.2	136.7 ± 6.9	176.6 ± 7.3	+18.8 ± 1.7	−19.2 ± 0.9	3.20
TiO_2_-Ag (5%)	9.3 ± 1.1	8.9 ± 1.3	153.4 ± 9.8	189.5 ± 8.1	+17.3 ± 0.8	−19.7 ± 1.4	3.15

^#^Particle size and zeta potential in solution were measured by ZetaSizer Nano-HT (Malvern). CDMEM: complete Dulbecco’s modified eagle media (DMEM + 10% fetal bovine serum). DI: deionized water. TEM: transmission electron microscopy. XRD: X-ray diffraction.

### Cytotoxicity

**Figure 5:**
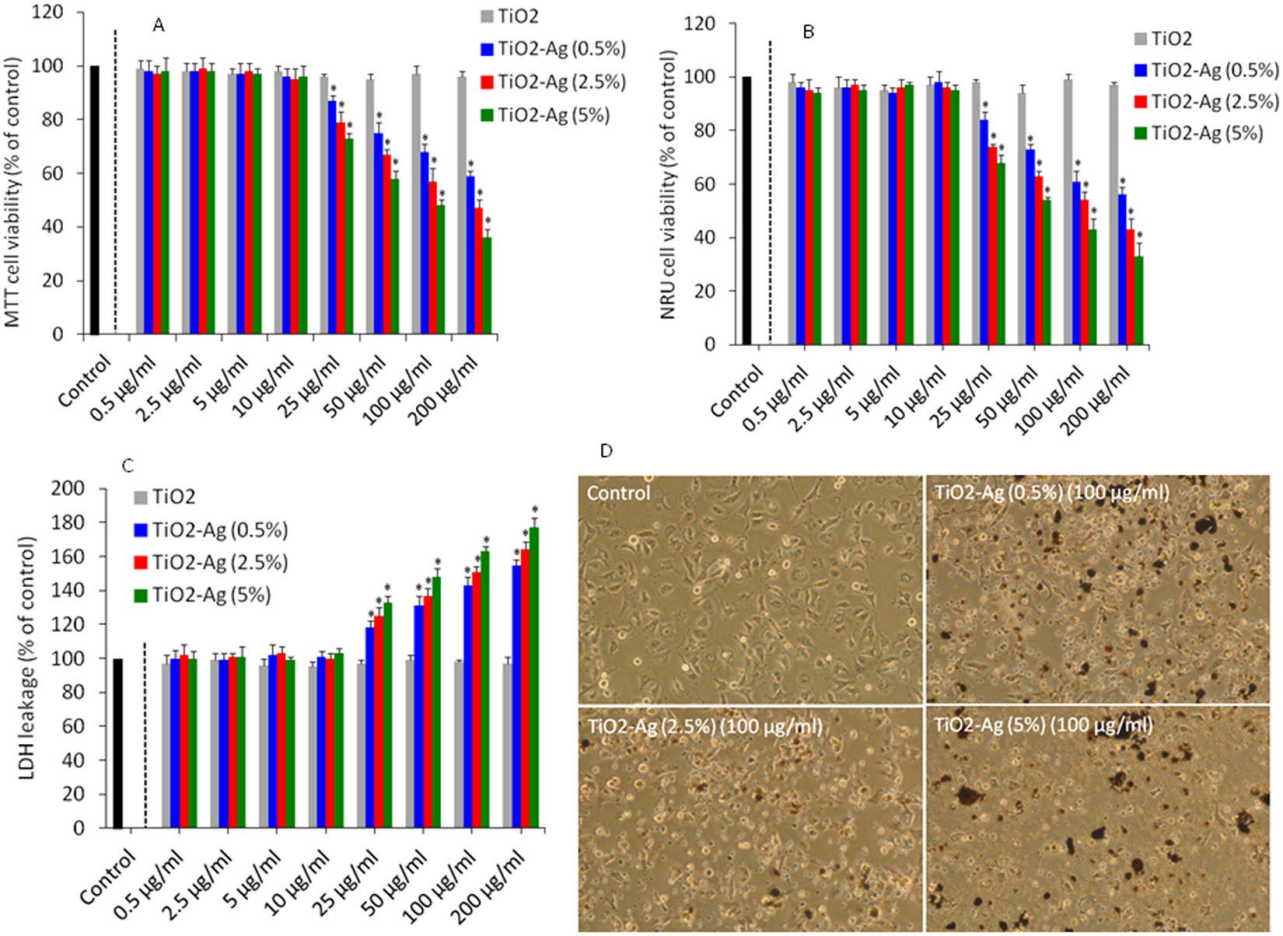
Cytotoxic response of pure and Ag-doped TiO_2_ NPs in HepG2 cells. (**A**) MTT cell viability. Cells were treated with 0.5–200 µg/ml of pure and Ag-doped TiO_2_ NPs for 24 h. (**B**) NRU cell viability. Exposure of NPs to cells was similar as in MTT assay. (**C**) Lactate dehydrogenase (LDH) enzyme leakage assay. Exposure of NPs to cells was similar as in MTT assay. Data represented are mean ± SD of three identical experiments made in three replicate. *Significant difference as compared to control (p < 0.05). (**D**) Cell morphology after exposure to Ag-doped TiO_2_ NPs at a concentration of 100 µg/ml for 24 h.

Human liver cancer (HepG2) cells were treated with different concentrations (0.5–200 µg/ml) of pure and Ag-doped TiO_2_ NPs for 24 h and cell viability was measured by MTT and NRU assays. Both parameters serve as sensitive and integrated tools to measure the cell integrity and cell proliferation inhibition^49,67^. The MTT assay was used to evaluate the mitochondrial function while NRU assay represents the lysosomal activity. Both MTT and NRU data showed that Ag-doped TiO_2_ NPs reduced the viable number of cells dose-dependently in the concentration range of 25–200 µg/ml. Besides, cell viability decreases with increasing concentrations of Ag dopant (Fig. 5A and B). On the other hand, pure TiO_2_ NPs did not reduce the viability of HepG2 cells.

LDH enzyme leakage in culture medium from cells is also an indicator of NPs penetration into cells^68^. A plenty of studies have shown that LDH level increases in culture medium after exposure to NPs^37,69^. Our results also demonstrated that Ag-doped TiO_2_ NPs induced LDH leakage and incremental Ag-doping resulted in higher leakage of LDH enzyme (Fig. 5C). However, pure TiO_2_ NPs did induce LDH leakage in HepG2 cells. To support LDH data we further studied the cellular uptake of pure and Ag-doped TiO_2_ in HepG2 cells by IC-MS. After exposure of 100 µg/ml pure and Ag-doped (0.5–5%) TiO_2_ NPs for 24 h, ICP-MS analysis showed the presence of Ti and Ag elements in HepG2 cells (Supplementary Fig. S2).

We further examined the morphology of HepG2 cells after exposure to Ag-doped (0.5–5%) TiO_2_ NPs at a concentration of 100 µg/ml for 24 h. Results demonstrated low cell density and rounding of cells after exposure to Ag-doped TiO_2_ NPs as compared to the controls (Fig. 5D). Similar to cell viability and LDH leakage results, morphology data showed that cytotoxic response of TiO_2_ NPs increases with increasing the amount of Ag-doping. Our previous study also reported that Zn-doped TiO_2_ NPs induced cytotoxicity in human breast cancer (MCF-7) cells^49^. Other studies have also shown that metal ions doping tunes the cytotoxic response of semiconductor metal oxide NPs^62,70,71^. These results were according to other reports demonstrating that pure TiO_2_ NPs did not induce cytotoxicity in different types of human cells^72,73^.

### Apoptosis

**Figure 6:**
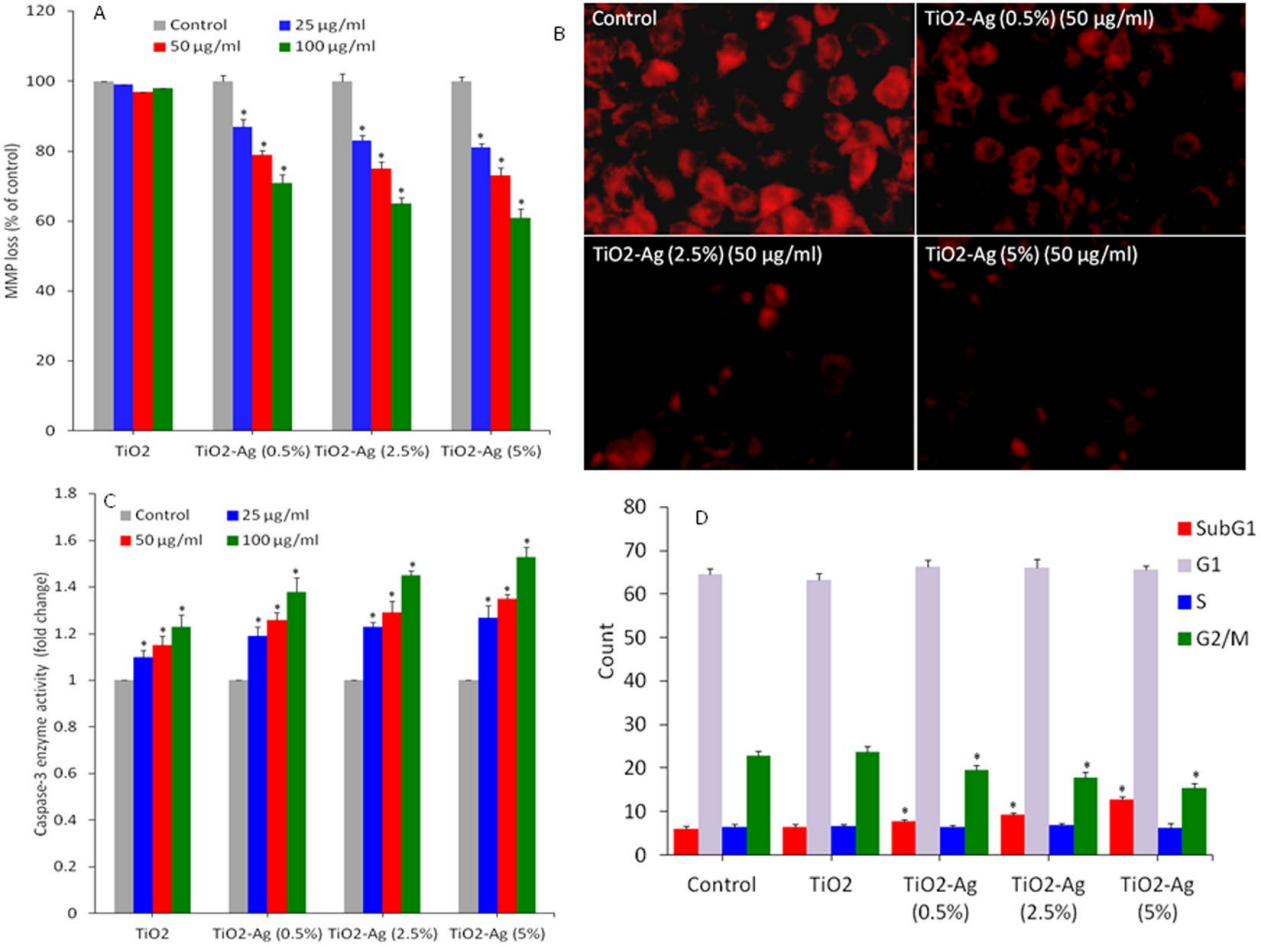
Apoptotic response of pure and Ag-doped TiO_2_ NPs in HepG2 cells. (**A**) Quantitative analysis of MMP. The MMP level was determined by Rh123 fluorescence probe. Cells were treated with 25, 50 & 100 µg/ml of pure and Ag-doped TiO_2_ NPs for 6 h. (**B**) Fluorescent microscopic images of Rh123 in treated and control cells. (**C**) Caspase-3 enzyme activity. Exposure of NPs to cells was similar as in MMP assay. (**D**) Cell cycle phases (SubG1, G1, S, and G2/M) of HepG2 cells after exposure to 100 µg/ml of pure and Ag-doped TiO_2_ NPs for 24 h. Cell cycle phases were examined by propidium iodide (PI) probe. Data represented are mean ± SD of three identical experiments made in three replicate. *Significant difference as compared to control (p < 0.05).

Apoptosis is known as a distinct mode of programmed cell death that involves the elimination of genetically damaged cells. Apoptotic cell death occurs as a defense mechanism when cellular DNA is damaged beyond the repair^74^. We studied the MMP level, caspase-3 enzyme activity and cell cycle as markers of apoptosis in HepG2 cells against pure and Ag-doped TiO_2_ NPs exposure. MMP level in HepG2 cells were measured after exposure to pure and Ag-doped TiO_2_ NPs at the concentration of 25–100 µg/ml for 6 h. MMP level was assayed using Rh-123 fluorescent probe. Quantitative data indicated that Ag-doped TiO_2_ NPs caused MMP loss in a dose-dependent manner (Fig. 6A). Fluorescence microscopy images also showed that the brightness of red intensity was decreases with increasing the concentration of Ag-doping (Fig. 6B). Caspase genes are activated during the process of cell death and are known to play critical roles in apoptotic pathway. Studies have shown that caspase-3 gene is imperative for genetic damage and programmed cell death^75^. Our results demonstrated that Ag-doped TiO_2_ NPs induced caspase-3 enzyme activity dose-dependently. Besides, caspase-3 enzyme activity was increases with increasing the level of Ag-doping (Fig. 6C). We further studied the cell cycle progression against pure and Ag-doped TiO_2_ NPs exposure. It is known that cells with damaged DNA accumulated in gap1 (G1), DNA synthesis (S) or in gap2/mitosis (G2/M) phase. Cells with irreversible damage undergo apoptosis, giving rise to accumulation of cells in sub-G1 phase. Flow-cytometric data demonstrated the induction of apoptosis in HepG2 cells upon exposure to Ag-doped TiO_2_ NPs exposure (Fig. 6D). The Ag-doped (5%) TiO_2_ NPs (100 µg/ml for 24 h) resulted in the appearance of a significant 12.8% cells in the sub-G1 phase than those of 6.1% of untreated control cells. A significant decline in G2/M phase was also evident in Ag-doped TiO_2_ NPs treated cells. Similar to cytotoxicity results, pure TiO_2_ NPs did not induce apoptosis in HepG2 cells.

### Oxidative stress

**Figure 7:**
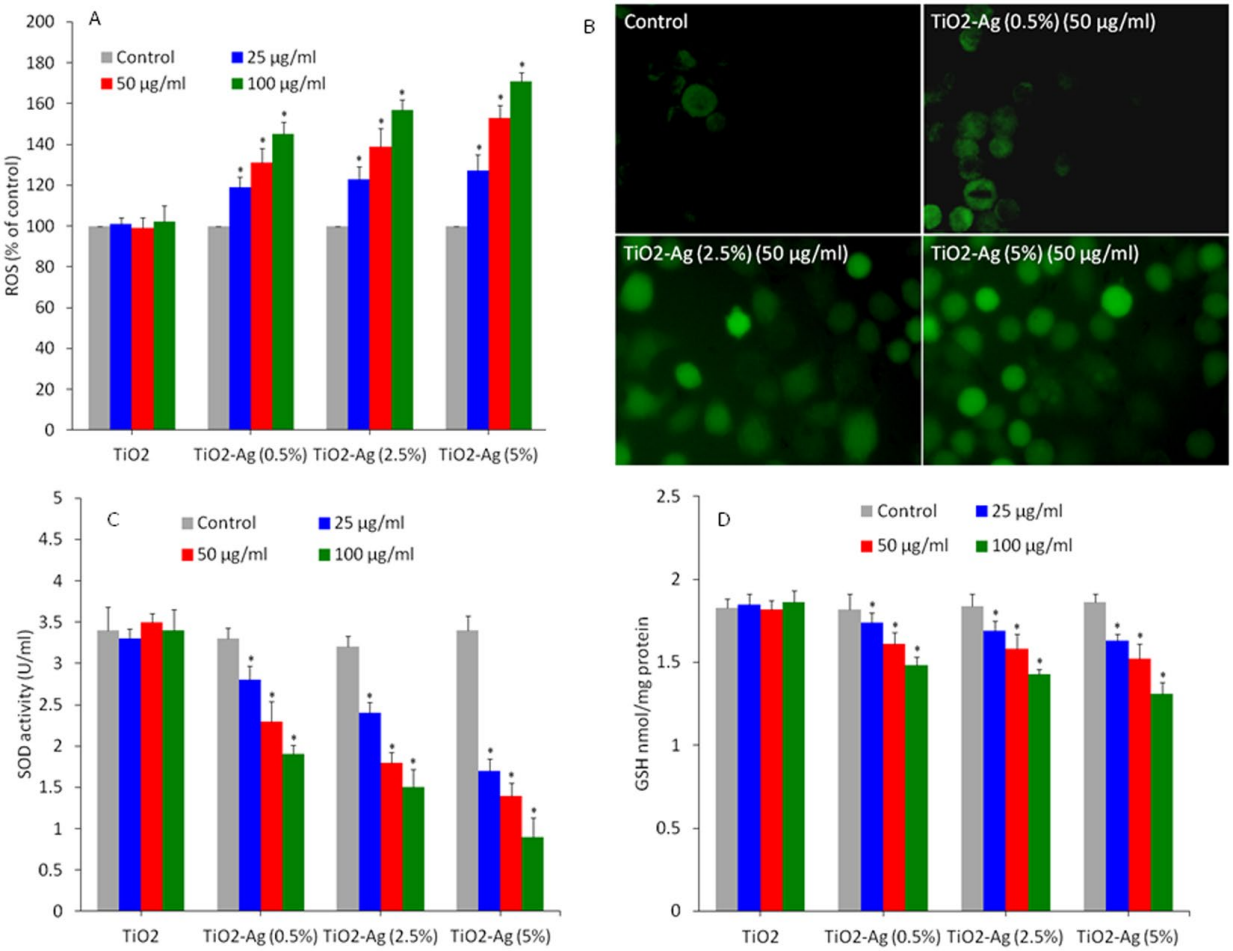
Oxidative stress response of pure and Ag-doped TiO_2_ NPs in HepG2 cells. (**A**) Quantitative assay of ROS level. Generation of intracellular ROS level was measured by 2,7-dichlorofluorescin diacetate (DCFH-DA) fluorescence-based assay. Cells were treated with 50, 100 & 200 µg/ml of pure and Ag-doped TiO_2_ NPs for 6 h. (**B**) Fluorescent microscopic images of ROS in treated and control cells. (**C**) SOD enzyme activity. Exposure of NPs to cells was similar as in ROS assay. (**D**) GSH level. Exposure of NPs to cells was similar as in ROS assay. Data represented are mean ± SD of three identical experiments made in three replicate. *Significant difference as compared to control (p < 0.05).

Oxidative stress has been played a critical role in the cytotoxic response of a number of NPs whether by the extreme generation of oxidants (e.g. ROS) or by reduction of antioxidants (e.g. GSH)^52,64,76^. Evidence are rapidly increasing that manipulation of intracellular ROS production can be utilized in killing of cancer cells without much affecting the normal cells^36,39^. Our earlier studies have shown that semiconductor nanoparticles such ZnO have potential to selectively kill cancer cells via ROS generation while sparing the normal cells^37,38,52^. In the present study, we further investigated the regulation of oxidative stress markers (ROS, SOD & GSH) in HepG2 cells upon exposure to 25, 50 and 100 μg/ml of pure and Ag-doped TiO_2_ NPs for 6 h. Intracellular ROS generation was assessed by DCFDA fluorescent probe. ROS such as superoxide anion (O_2_^•−^), hydroxyl radical (HO^•^) and hydrogen peroxide (H_2_O_2_) elicit a variety of physiological and cellular events including DNA damage and apoptosis^76,77^. Quantitative results demonstrated that ROS level was increases dose-dependently and proportional to the amount of Ag-doping in TiO_2_ NPs (Fig. 7A). Fluorescence microscopy images also supporting that the brightness of green probe was higher in Ag-doped TiO_2_ NPs in comparison to controls (Fig. 7B). Although, pure TiO_2_ NPs did not induce ROS production in HepG2 cells. Superoxide dismutase (SOD) enzyme is acting as front liner in antioxidant defense system. This enzyme catalyses the dismutation of highly reactive superoxide (O_2_^•−^) anion into hydrogen peroxides (H_2_O_2_). We observed dose-dependent reduction in SOD enzyme activity and proportional to the Ag-doping (Fig. 7C). On the other hand, pure TiO_2_ NPs did not affect the activity of SOD enzyme. Higher production of intracellular ROS leads to oxidize the cellular biomolecules such as glutathione (GSH), which plays a critical role in maintaining the redox homeostasis through its antioxidant activity. We also found that Ag-doped TiO_2_ NPs induced GSH depletion in dose-dependent manner and proportional to the amount of Ag-doping (Fig. 7D).

### Ag-doped TiO2 NPs induced cytotoxicity in HepG2 cells via oxidative stress

**Figure 8:**
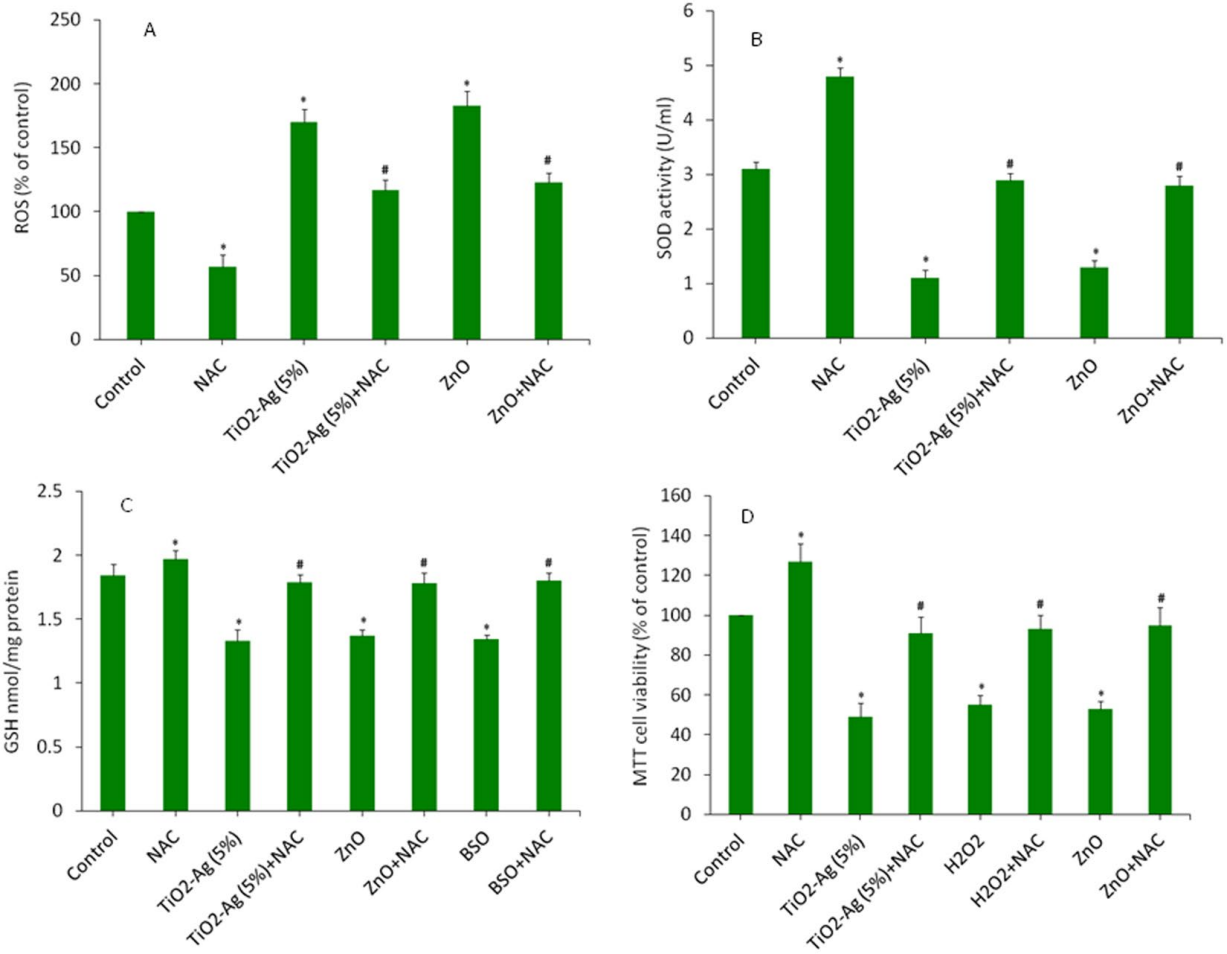
Ag-doped TiO_2_ NPs induced cytotoxicity was mediated via oxidative stress. Antioxidant N-acetylcysteine (NAC) effectively prevented the oxidative stress and cytotoxicity induced by Ag-doped TiO_2_ NPs in HepG2 cells. Cells were treated with Ag-doped (5%) TiO_2_ NPs at the concentration of 100 µg/ml in the presence or absence of NAC (10 mM) for 6 or 24 h. The ZnO NPs, H_2_O_2_ or BSO were used as positive controls. (**A**) ROS level, (**B**) SOD activity, (**C**) GSH level and (**D**) MTT cell viability. Data represented are mean ± SD of three identical experiments made in three replicate. *Significant difference as compared to the control (p < 0.05). ^#^Significant effect of NAC against NPs and positive controls (p < 0.05).

In this section, we explored the role of ROS and oxidative stress in cytotoxic response of Ag-doped (5%) TiO_2_ NPs in HepG2 cells (Fig. 8). The HepG2 cells were treated with Ag-doped (5%) TiO_2_ NPs with or without N-acetyl-cysteine (NAC) or buthionine sulphoximine (BSO). We have also used ZnO NPs or H_2_O_2_ as positive controls. Results demonstrated that NAC efficiently averted the ROS generation and SOD depletion caused by Ag-doped TiO_2_ NPs, or ZnO NPs (Fig. 8A and B). BSO was used as positive control for GSH depletion. Besides, NAC exposure restored the GSH in cells treated with Ag-doped TiO_2_ NPs or BSO (Fig. 8C). At last, we also found that co-exposure of NAC, effectively abolished the cytotoxicity induced Ag-doped TiO_2_ NPs, ZnO NPs or H_2_O_2_ (Fig. 8D). Altogether, these results suggested that oxidative stress could be one of the potential mechanisms of toxicity induced by Ag-doped TiO_2_ NPs in human liver cancer (HepG2) cells.

### Cytotoxicity and oxidative response of Ag-doped TiO2 NPs in human lung and breast cancer cells

**Figure 9:**
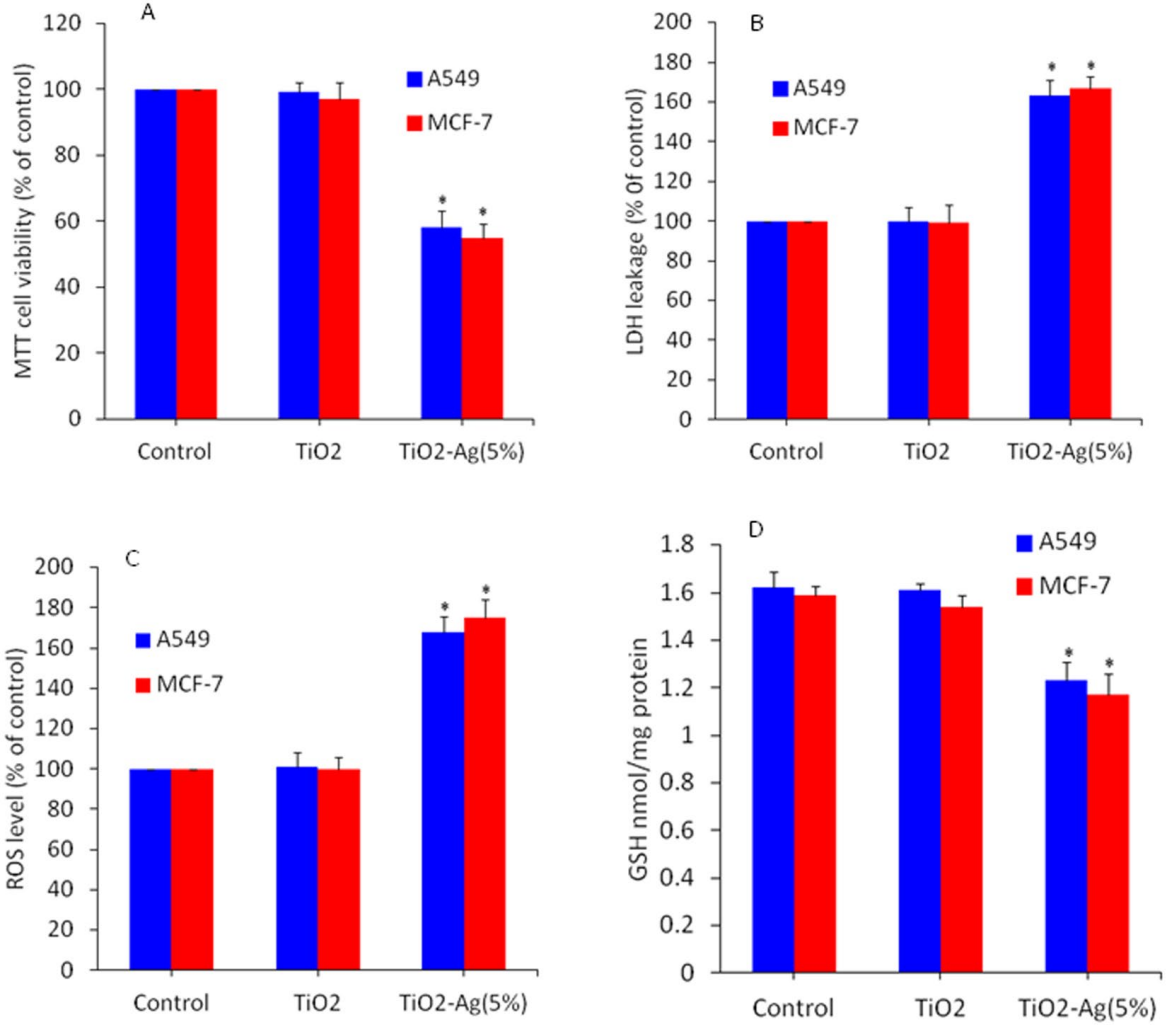
Ag-doped TiO_2_ NPs induced cytotoxicity and oxidative stress in human lung (A549) and breast (MCF-7) cancer cells. (**A**) MTT cell viability assay. Cells were treated with 100 µg/ml of pure and Ag-doped (5%) TiO_2_ NPs for 24 h. (**B**) LDH leakage assay. Exposure of NPs to cells was similar as in MTT assay. (**C**) Intracellular ROS level. Cells were treated with 100 µg/ml of pure and Ag-doped TiO2 NPs for 6 h. (**D**) GSH level. Exposure of NPs to cells was similar as in ROS assay. Data represented are mean ± SD of three identical experiments made in three replicate. *Significant difference as compared to control (p < 0.05).

To avoid cell type specific response we have also employed human breast (MCF-7) and lung (549) cancer cells to see the effect of Ag-doped (5%) TiO_2_ NPs. Cytotoxicity endpoints (MTT & LDH assays) and oxidative stress markers (ROS & GSH levels) were assessed. We observed that like HepG2 cells, Ag-doped TiO_2_ NPs causes reduction in cell viability (Fig. 9A), LDH leakage (Fig. 9B), higher level of ROS (Fig. 9C) and depletion of GSH (Fig. 9D) in MCF-7 and A549 cells. However, pure TiO_2_ NPs did not cause toxicity to both types of ells. These results are suggesting that the potential mechanism of toxicity induced by Ag-doped TiO_2_ NPs in A549 and MCF- cells was comparable to HepG2 cells.

### Amount of Ag present in Ag-doped TiO_2_ NPs did not cause cytotoxicity alone to human cancer cells

**Figure 10:**
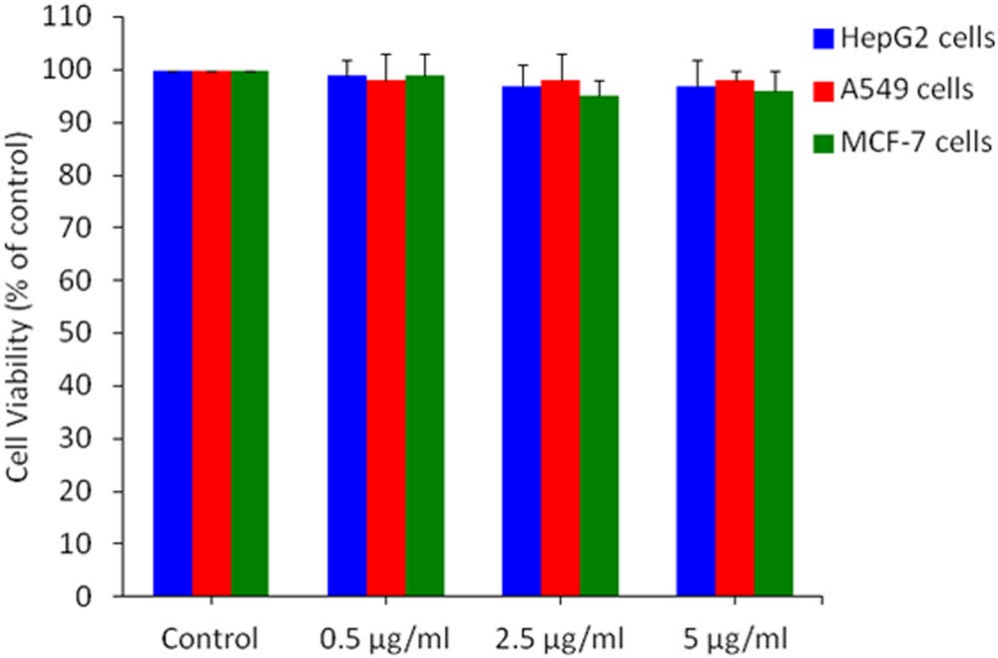
Amount of Ag present in Ag-doped TiO_2_ nano-composite did not induced toxicity alone to HepG2, A549 and MCF-7 cancer cells. Cytotoxicity was measured by MTT cell viability assay. Cells were exposed to 0.5, 2.5 and 5 µg/ml of Ag NPs. This is the amount of Ag present in the 100 µg/ml of Ag-doped TiO_2_ NPs. Data represented are mean ± SD of three identical experiments made in three replicate.

We found that pure TiO_2_ NPs did not cause toxic effects to selected human cancer cell lines (HepG2, A549 & MCF-7). However, Ag-doped TiO_2_ nano-complex induced toxicity to these cells. To make clear that observed toxic effect was due to exposure Ag-TiO_2_ nanocomplex not by Ag alone, we examine the effect of Ag NPs alone in these cell lines. We selected the 0.5, 2.5 & 5 µg/ml of Ag NPs for cytotxicity assays. These amounts of Ag present in 100 µg/ml solution of Ag-doped (0.5, 2.5 & 5%) TiO_2_ NPs. We exposed HepG2, A549 and MCF-7 cells with Ag NPs at the concentration of 0.5, 2.5 and 5 µg/ml for time period of 24 h. After the completion of exposure time, cell viability was measured by MTT assay. Results have shown that selected concentration of Ag NPs were not able to exert cytotoxicity to all three types of cancer cells (Fig. 10). These results indicated that Ag-TiO_2_ nanocomplex was responsible for cytotoxicity, apoptosis and oxidative stress in cancer cells neither Ag nor TiO_2_ alone.

### Ag-doped TiO_2_ NPs were benign to normal cells

**Figure 11:**
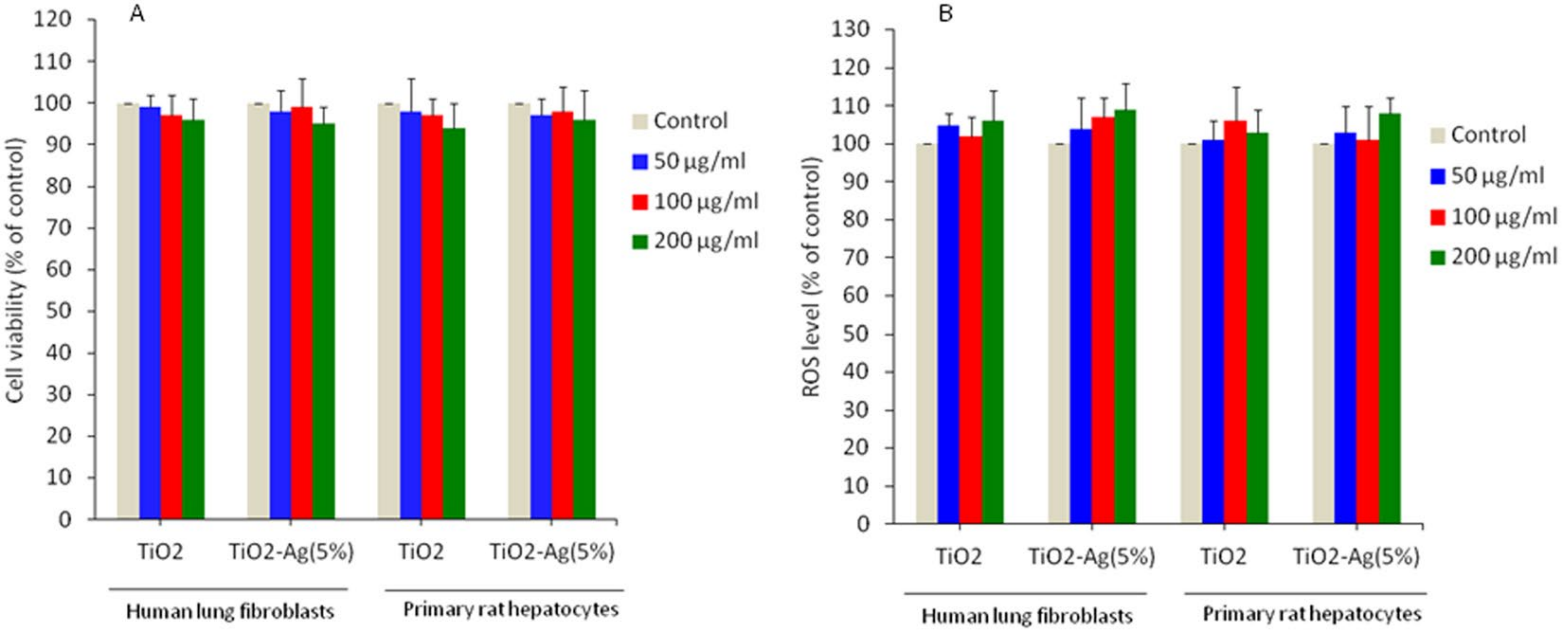
Ag-doped TiO_2_ NPs did not induce toxicity to non-cancerous cells. (**A**) MTT cell viability assay in human lung fibroblasts (IMR-90) and primary rat hepatocytes. Cells were treated with 100 µg/ml of pure and Ag-doped (5%) TiO_2_ NPs for 24 h. (**B**) Intracellular ROS level. Cells were treated with 100 µg/ml of pure and Ag-doped (5%) TiO_2_ NPs for 6 h. Data represented are mean ± SD of three identical experiments made in three replicate.

To see the benign nature of Ag-doped TiO_2_ NPs toward normal cells, we have examined the effect of Ag-doped (5%) TiO_2_ NPs on human lung fibroblasts (IMR-90) and primary rat hepatocytes. Results demonstrated that Ag-doped TiO_2_ NPs did induce cytotoxicity and oxidative stress in both types of normal cells (Fig. 11A and B). Other studies have also reported the benign nature of TiO_2_ NPs^78–80^. These results suggested that Ag-doped TiO_2_ NPs have inherent selective toxicity nature towards cancer cells while posing no effect to normal cells. In previous studies, we also found that ZnO and Al-doped ZnO NPs have the inherent selective killing nature towards cancer cells without posing much effect to normal cells^37,38^. These results suggested that Ag-doped TiO_2_ NPs has anticancer activity. Preferential cancer cells killing ability of metal-based NPs are being explored at laboratory level^38,39,71,81,82^.

## Conclusions

We found that Ag-doped TiO_2_ NPs induced toxicity in human liver cancer (HepG2) cells *via* oxidative stress. The toxic intensity of Ag-doped TiO_2_ NPs was increases with the incremental of Ag level. This is possibly due to the tuning of size and band gap of TiO_2_ NPs by Ag-doping. Furthermore, Ag-doped TiO_2_ NPs were also induced toxicity to human lung (A549) and breast (MCF-7) cancer cells. On the other hand, Ag-doped TiO_2_ NPs spare the normal human lung fibroblasts (IMR-90) and primary rat hepatocytes. Altogether, our data suggested that Ag-doped TiO_2_ NPs selectively kill cancer cells while sparing the normal cells. This preliminary report on selective toxicity of Ag-doped TiO_2_ nano-complex toward cancer cells warranted further extensive research on various types of cancer and normal cells along with *in vivo* models.

## Electronic supplementary material

Supplementary Information
