# Supplementary material for: Ag-doping regulates the cytotoxicity of TiO2 nanoparticles via oxidative stress in human cancer cells
Source: Sci Rep. 2017 Dec 15;7:17662. doi: 10.1038/s41598-017-17559-9 (PMC5732217; doi:10.1038/s41598-017-17559-9)
Supplement: Supplementary file 1 — Supplementary Information [file 41598_2017_17559_MOESM1_ESM.pdf]

Supplementary Information

Ag-doping regulates the cytotoxicity of TiO_2 nanoparticles *via* oxidative stress in human cancer cells

Maqusood Ahamed [1,*] M.A. Majeed Khan [1], Mohd Javed Akhtar [1], Hisham A. Alhadlaq [1,2], Aws Alshamsan [1,3],

[1] King Abdullah Institute for Nanotechnology, King Saud University, Riyadh, Saudi Arabia

[2] Department of Physics and Astronomy, College of Science, King Saud University, Riyadh, Saudi Arabia

[3] Nanomedicine Research Unit, Department of Pharmaceutics, College of Pharmacy, King Saud University, Riyadh, Saudi Arabia

[*]Corresponding author:

Dr. Maqusood Ahamed

Assistant Professor

King Abdullah Institute for Nanotechnology

King Saud University

Riyadh 11451, Saudi Arabia

Phone: +966114698781

Email: maqusood@gmal.com; mahamed@ksu.edu.sa

A

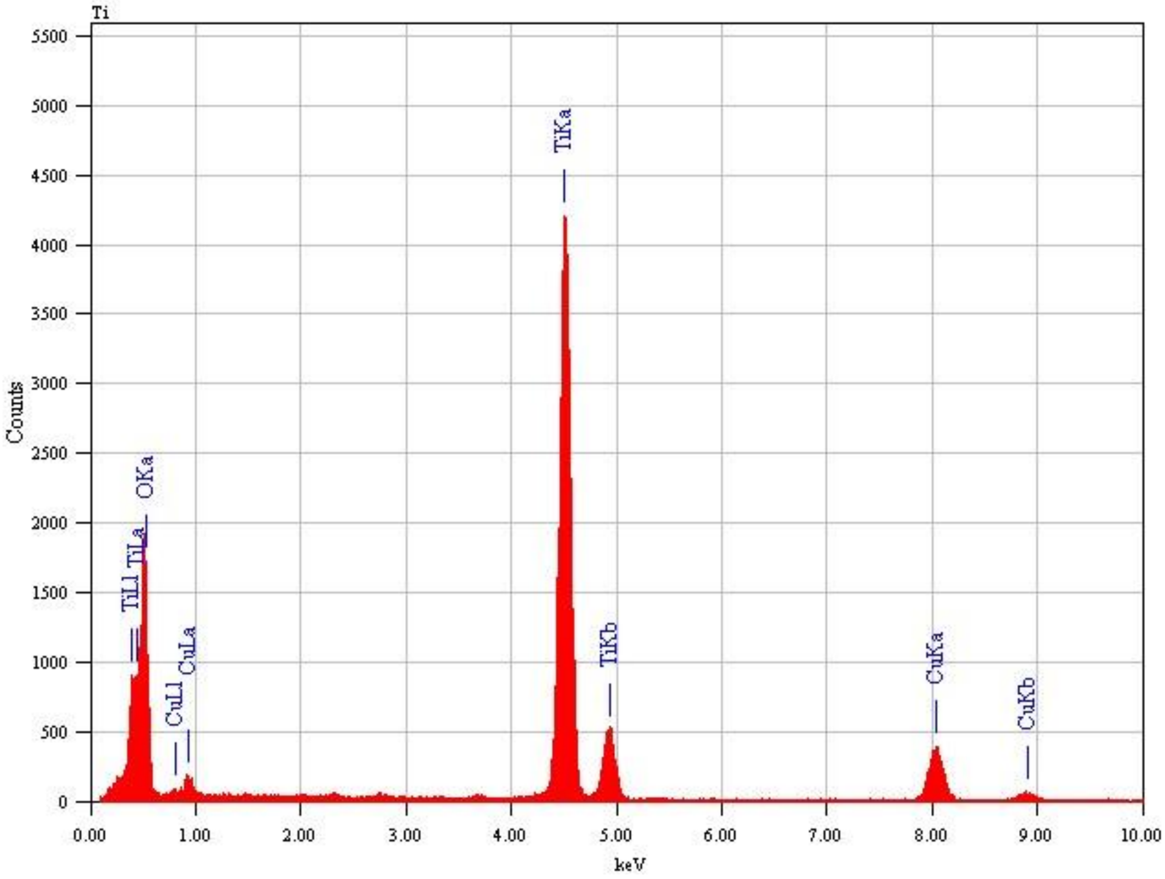

B

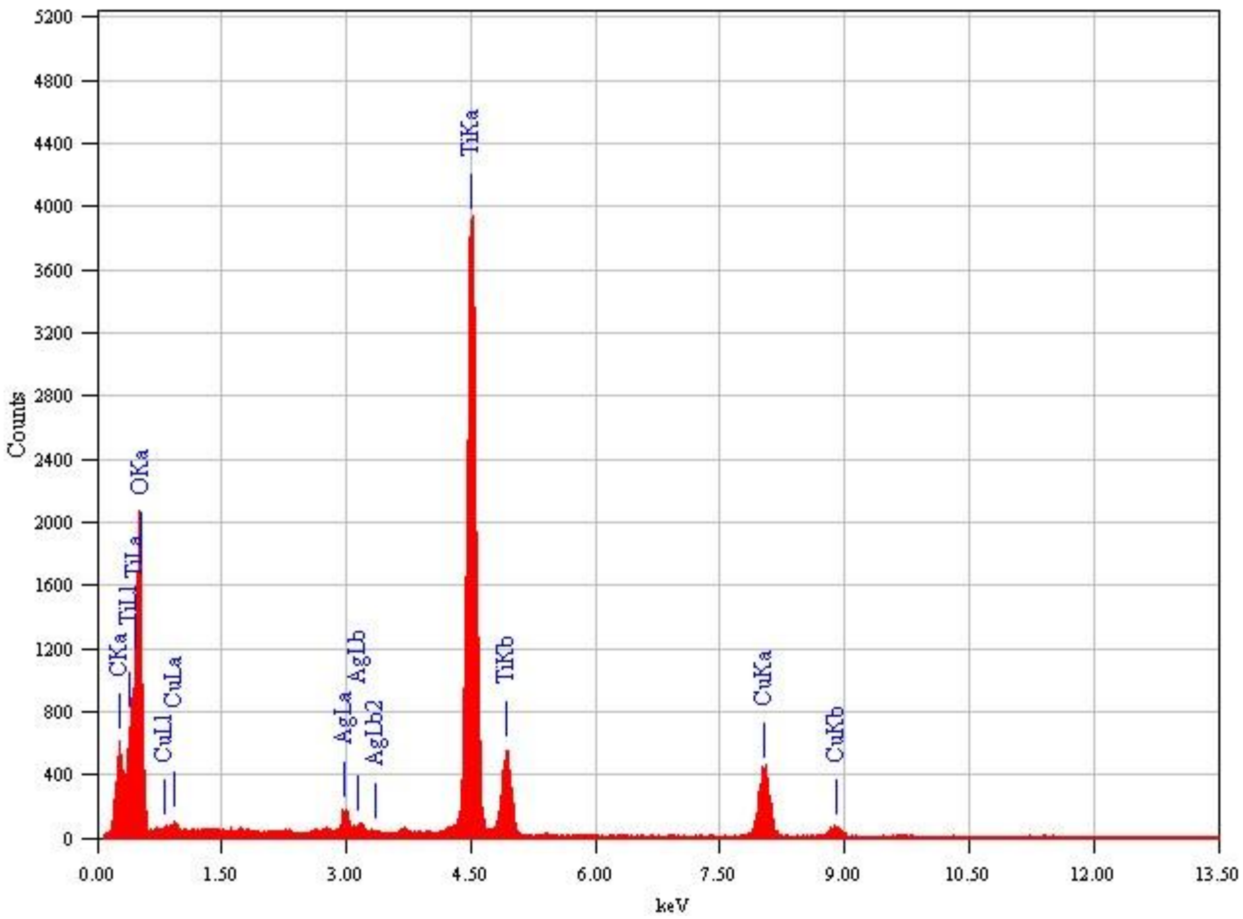

Supplementary Figure S1: Energy dispersive X-ray spectroscopy (EDS) analysis of pure and Ag-doped TiO_2 NPs. It is clear from these images that Ti and O were the main elemental species in pure TiO_2 NPs while additional Ag peaks were observed in Ag-doped TiO_2 NPs. No other element impurity was detected. The peaks of Cu and C observed in the spectra are attributed to the carbon-coated copper TEM grid. (A) EDS spectrum of pure TiO_2 NPs and (B) Ag-doped TiO_2 NPs.

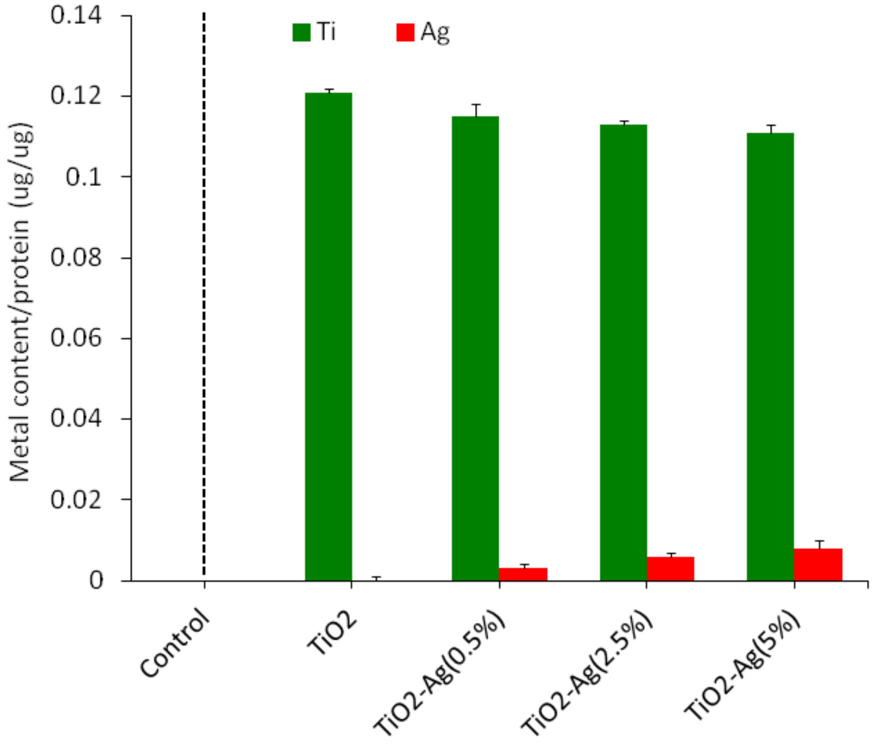

Supplementary Figure S2: ICP-MS analysis to measure the cellular Ti and Ag content in HepG2 cells. Cells were treated with 100 µg/ml of NPs for 24 h, and untreated cells were used as a control. After PBS washing, cells were harvested using 0.05% trypsin and the cell suspension digested with concentrated nitric acid at 90 °C for 4 h. The well-digested solution was used for ICP-MS measurement.
